# Serum Levels of Growth-Associated Protein-43 and Neurotrophin-3 in Childhood Epilepsy and Their Relation to Zinc Levels

**DOI:** 10.1007/s12011-022-03213-7

**Published:** 2022-03-29

**Authors:** Ali Helmi Bakri, Mohammed H. Hassan, Ahmed El-Abd Ahmed, Ghallab Alotaibi, Pola Rafat Halim, Ahmed Alamir Mahmoud Abdallah, Nagwan I. Rashwan

**Affiliations:** 1 Department of Pediatrics, Faculty of Medicine, South Valley University, Qena, Egypt; 2 Department of Medical Biochemistry, Faculty of Medicine, South Valley University, Qena, 83523 Egypt; 3 Department of Pharmaceutical Sciences, College of Pharmacy, Shaqra University, Al-Dawadmi Campus, Shaqra, Saudi Arabia; 4 Medical Biochemistry Department, Faculty of Medicine, Sohag University, Sohag, Egypt

**Keywords:** Growth-associated protein-43, Neurotrophin-3, Total antioxidant capacity, Zinc, Childhood epilepsy

## Abstract

**Background:**

Epilepsy is one of the most common neurological disorders, and it places a significant economic strain on the healthcare system around the world. Although the exact mechanism of epilepsy has yet to be illustrated, various pathogenic cascades involving neurotransmitters and trace elements have been reported. We aimed to investigate the serum levels of growth-associated protein-43 (GAP-43) and neurotrophin-3 (NT-3) among cohort of Egyptian children with epilepsy and correlate these biomarkers with their zinc levels.

**Methods:**

This case–control study included 50 pediatric patients with epilepsy who were comparable with 50 controls. Neurological assessment and electroencephalogram (EEG) were done to all included children. Biochemical measurements of serum GAP-43 and NT-3 using enzyme linked immunosorbent assays (ELISA), and total antioxidant capacity (TAC) and zinc using colorimetric assays, were performed to all participants.

**Results:**

There was significantly frequent positive parental consanguinity among cases with significantly frequent generalized onset seizures (94%) than simple partial seizure (6%). There were significantly lower serum GAP-43 and zinc levels with significantly higher TAC among cases vs. the controls, *p*˂0.05 for all. There was no significant difference in the serum levels of NT-3 among epileptic children vs. the controls, *p* = 0.269. Serum Zn was positively correlated with GAP-43 level among epileptic children (*r* = 0.381, *p* = 0.006). Serum GAP-43 in diagnosing childhood epilepsy at cut-off point ≤ 0.6 ng/mL showed 78% sensitivity, 62% specificity, positive predictive value (*PPV*) = 50.6%, negative predictive value (*NPP*) = 84.9% with *AUC* = 0.574.

**Conclusion:**

GAP-43 can be considered a sensitive good negative biomarker in childhood epilepsy which correlated positively with the zinc status.

## Introduction

Epilepsy is a major health concern that affects around 70 million people globally, with a much higher incidence in children [1, 2]. It is regarded as one of the most common neurological disorders, with a significant economic strain on the worldwide healthcare system [3]. Childhood epilepsy is linked to neurological, mental health, and cognitive issues, all of which have a significant negative impact on quality of life [4]. According to statistics, the majority of children suffer their first epileptic episodes before the age of three years [5], highlighting the need for early intervention. Furthermore, a considerable body of information suggests that about half of epileptic patients do not respond to pharmacological treatment and continue to have seizures [6]. As a result, it is critical to completely comprehend the mechanisms of disease onset and progression in order to find innovative ways to build early diagnostic tools and preventative assessments.

Although the specific mechanism of epilepsy has yet to be discovered, various pathogenic cascades involving neurotransmitters and trace elements have been discovered. Growth-associated protein-43 (GAP-43), a marker of newly formed synapses and a trace element of zinc, and neurotrophin-3 (NT-3) have been demonstrated to play important roles in epileptic episodes [7–9]. These cellular and molecular alterations have a significant impact on epilepsy development. Inadequate evidence suggests that GAP-43 plays a significant role in epileptogenesis-induced synaptic plasticity in neural networks [10–14].

Neurotrophin-3 appears to have a vital function in fostering neuronal differentiation and boosting neurite outgrowth in the developing central nervous system, according to growing evidence [15, 16]. Furthermore, inconclusive findings have suggested that NT-3 expression is linked to epileptic activity.

Furthermore, changes in zinc trace element have been shown to influence epileptogenesis and contribute considerably to the occurrence of epileptic seizures [17]. This conclusion is based on the fact that neuronal excitement has a significant impact on zinc concentration [18]. It is worth noting that knocking down zinc transporters has been proven to improve susceptibility to kainate-induced seizures in studies [19], emphasizing the importance of zinc during epileptic episodes Animal studies have shown that zinc has neuromodulatory effects in epileptogenesis [20].

Given the fact that significant cellular and molecular changes of GAP-43 and NT-3 during epileptic episodes have been fully explored individually, however, their association with zinc has not been investigated. Therefore, we investigate the expression profiles of neurotrophin-3 and growth-associated protein-43 in children with epileptic seizures, to determine their potential role in epileptic seizures pathogenesis and diagnosis. We also investigate their associations with zinc and total antioxidant capacity in these children.

## Materials and Methods

### Study Design and Participants

The current case–control study comprised 50 pediatric patients with newly diagnosed epileptic seizures who were admitted to the Pediatrics Department (hospital ward) of Qena University Hospital, South Valley University, Egypt. The included newly diagnosed epileptic children encountered previous two unprovoked seizures and suffered a new seizure with the diagnosis of epilepsy and before starting anti-epileptic medications, blood samples were taken for assays of the studied biochemical markers to avoid any confounding factors regarding their serum levels. A control group of 50 unrelated healthy children from Outpatient Pediatric Clinics was also chosen, with age, sex, and BMI matching the included patients. The study was authorized by the Ethics Committee of the Faculty of Medicine, South Valley University, Qena, Egypt (Ethical approval code: SVU-MED-BIO-2021–12), and was conducted in accordance with the Declaration of Helsinki. The parents or caregivers of each infant enrolled in the study have given their informed written consent. The study duration was one year from July 1, 2020, to August 30, 2021.

### Patients' Selection Criteria

Infants and children with epilepsy were enrolled in the current study, but those with underlying central nervous system infections or abnormalities, febrile seizures, or those taking antiepileptic drugs, as well as those with chronic diseases (tuberculosis, malignancies, renal impairment, metabolic or hematological diseases) were all excluded. Infants with electrolyte disturbances, well-defined inborn metabolic defects, and abnormal computed topography suggestive of severe brain diseases or trauma were also excluded from the study.

### Clinical Assessments and Data Collection

Parents or caregivers were asked about the nature and duration of seizures, the presence and duration of the post-ictal phase, recent infectious diseases or fevers, recent antibiotic therapy, other associated symptoms, immunization and vaccination history, parental consanguinity, family history of epilepsy, or neurologic diseases.

### Electroencephalogram “EEG” Recording

With the children seated in a comfortable chair, EEG recordings were made for 10 min while they were resting with their eyes open. For 20–30 min, a Nihon khoden 8-channels conventional EEG equipment (NIHON KOHDEN – MEB-9400) with a ten-twenty international electrode placement system and unipolar and bipolar montages was employed. On the site of each electrode, electrode gel was placed. The impedance between the electrode and the skin was kept below 5 kΩ. With linked ears, all measurements were taken preferentially. All of the EEG data was evaluated after the testing was completed to rule out artifacts including blinks, eye movements, and muscular activity. For analysis, a minimum of 75 s of artifact-free EEG was provided. The data was divided into 4-s epochs after artifacts were removed, and the magnitude of each frequency band in microvolts was determined using Fourier power spectrum analysis [21].

### Blood Samples and Biochemical Assays

Each participant had a total of 3 mL of peripheral blood venous samples collected (within 8 h after the epileptic seizure [22]) and evacuated into serum separator gel tubes, where the samples were clotted for 30 min at 37 °C before centrifugation for 15 min at 3,500 rpm. The separated sera were kept at − 80 °C until biochemical analysis in the form of:

A) Serum neurotrophin-3 and growth-associated protein-43 assays were measured by microplate ELISA reader (EMR-500, Labomed, Inc. Los Angeles, USA), using commercially available ELISA assay kits supplied by Chongqing Biospes Co., Ltd., and SinoGeneClon Biotech Co., Ltd., respectively, China. Catalog No. BZEK1442-48 and SG-15851 respectively, according to the manufacturer protocol [22].
B) Serum TAC and zinc were measured by spectrophotometer (Chem-7, Erba Diagnostics Mannheim GmbH, Germany), using commercially available colorimetric assay kits supplied by Biodiagnostics, and Spectrum Diagnostics respectively, Cairo, Egypt. Catalog No. TA 25 13, and 330,001 respectively [22].

### Statistical Analysis

For data entry and analysis, SPSS version 19 was used (Statistical Package for Social Science). Numbers, percentages, median, and inter-quartile range (IQR) were used for non-parametric data, while mean ± SD was used for parametric data. The Chi-square test and Fisher exact test were used to compare qualitative variables. For non-parametric data, the Mann–Whitney test was employed to compare two quantitative variables. In the instance of non-parametric data, Spearman correlation was used to determine the correlation between quantitative variables. The Medcalc Program was used to calculate the sensitivity, specificity, positive and negative predictive values. Statistical significance was defined as a *p* value of less than 0.05.

## Results

### Demographic and Clinical Data of the Study Groups

The current study included 50 pediatric patients with newly diagnosed epilepsy (25 males and 25 females); they were compared to 50 age and sex-matched healthy participants as a control group (20 male and 30 female). There was no significant difference between the two groups as regard age and sex (*p*˃0.05), indicating age and sex matching (Table 1). There was significantly higher frequency of positive parental consanguinity among cases (28%) compared to the controls (8%), *p*˂ 0.05 (Table 1).

**Table 1:** Demographic data of the study groups

Variables	Epilepsy (*n* = 50)	Controls (*n* = 50)	*p* value
Age (month)			
Median(IQ range)	36(18–72)	34 (17–70)	0.07
Body mass index ( kg/m^2^ Mean ± SD)	16.40 ± 2.13	16.53 ± 1.83	0.683
Sex			
Male	25 (50%)	20 (40%)	0.10
Female	25 (50%)	30 (60%)	
Parental consanguinity			
+ ve Consanguinity	14(28%)	4 (8.0%)	0.011*
− ve Consanguinity	36(72%)	46(92.0%)	

^*^Significant *p* value (*p*<0.05)

Among the included cases, generalized onset seizures were significantly more frequent (94%) than partial onset seizure (6%). Sleep and abnormal behavior was frequently occurring post-ictal manifestations among epileptic patients (60% and 16% respectively). There was delayed physical development in 26% and mental delay was observed in 38% of epileptic patients (Table 2). Twenty-three cases (46%) have normal EEG records, while 27 cases (54%) have epileptic focus (23 cases have generalized and 4 cases have focal epileptiform discharges) on their EEG records. Positive family history for epilepsy was present in 10 cases (20%) (Table 2).

**Table 2:** Clinical data of the included pediatric patients with epilepsy

Variables	Epileptic children (*n* = 50) No.,%
Physical development	
Delay	13(26%)
Normal	37(74%)
Mental development	
Delay	19(38%)
Normal	31(62%)
EEG record findings	
Generalized epileptiform discharge	23(46%)
Focal epileptiform discharge	4(8%)
Normal	23(46%)
Type of seizures	
Generalized onset seizure	47(94%)
Partial onset seizure	3(6%)
Duration of fit (min)	
Median(IQ range)	30(15–37.5)
Post-ictal phase manifestations	
Abnormal behavior	8(16%)
Drowsy	0(0%)
Sleep	30(60%)
Vomiting	3(6%)
No	9(18%)
Precipitating factors	
Chest infection	3(6%)
G.E	12(24%)
No	35(70%)
Family history of seizures	
Yes	10(20%)
No	40(80%)

### Profiles of Serum Levels of Neurotrophin-3, Growth-Associated Protein-43, Total Antioxidant Capacity, and Zinc Among the Study Groups

There were significantly lower median values and IQR of serum levels of GAP-43 among cases vs. the controls, *p* = 0.024. Additionally, there were significantly lower zinc serum values and significantly higher TAC among cases compared to the controls, *p* < 0.001 for both (Table 3), although there was higher median serum values of NT-3 among epileptic children vs. the controls, but not reach a significant difference, *p* = 0.269 (Table 3).

**Table 3:** Circulating growth associated protein-43, neurotropin-3, total antioxidant capacity, and zinc profile among the study groups

Studied specific biochemical markers	Epileptic children (*n* = 50)	Controls (*n* = 50)	*p* value
Serum growth associated protein-43 (ng/mL)			
Median(IQ range)	2.01 (0.61–4.48)	7.32 (0.76–25.25)	0.024*
Neurotropin-3 (ng/L)			
Median(IQ range)	70.96 (55.26–216.39)	36.55 (25.56–951.7)	0.269
Total antioxidant capacity (mmol/mL)			
Median(IQ range)	0.68 (0.6–0.99)	0.31 (0.3–0.45)	< 0.001*
Serum zinc (μmol/L)			
Median(IQ range)	5.2 (3.42–8.13)	16.54 (14.76–18.2)	< 0.001*

^*^Significant *p* value (*p*˂0.05)

### Correlations Between the Studied Biochemical Markers Among Pediatric Patients with Epilepsy

**Fig.1:**
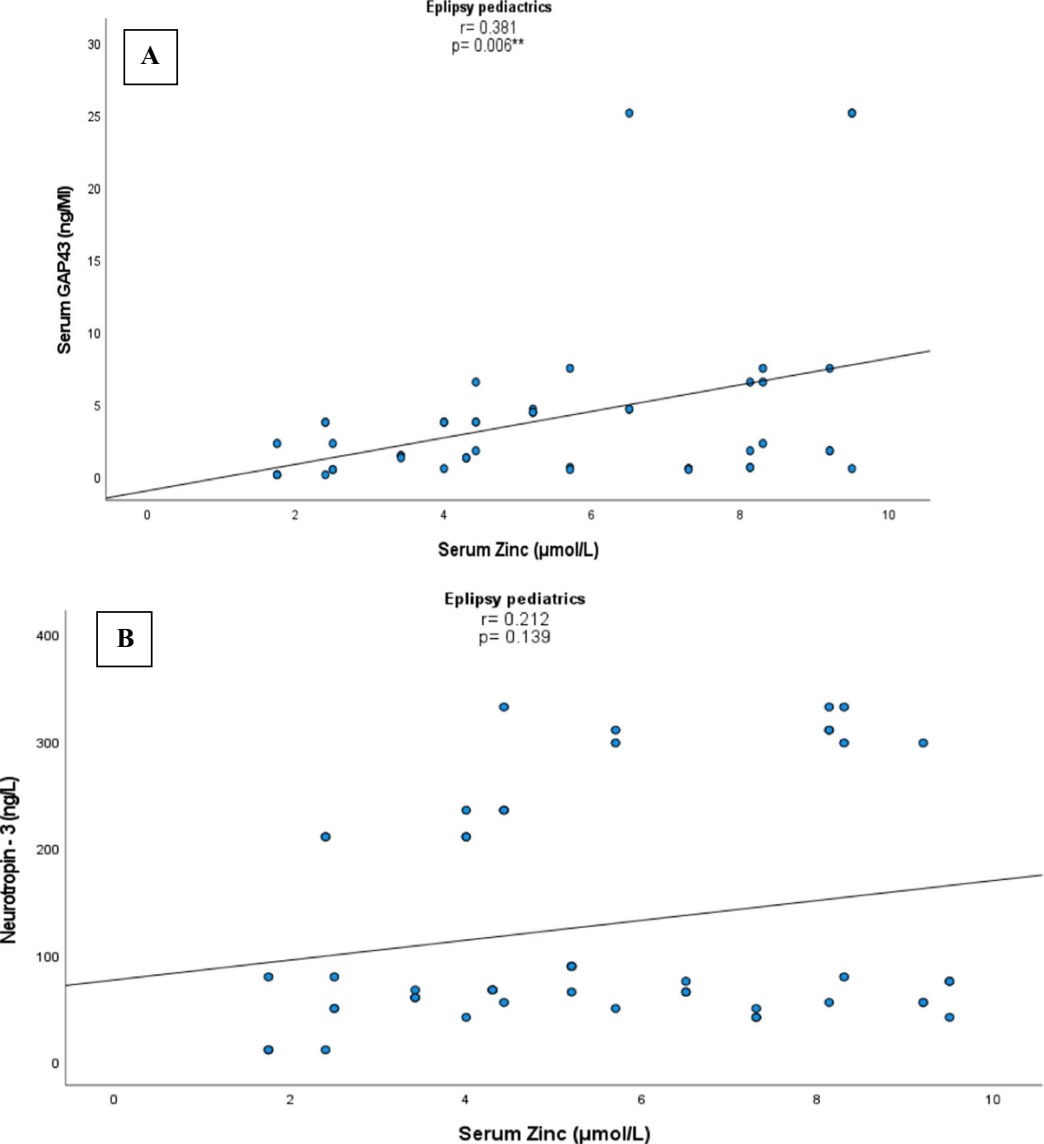
Correlations between serum zinc with both serum GAP43 and NT-3 among epileptic pediatric patients.** A**: showed positive correlations between serum GAP43 and zinc levels. **B**: showed lack of association between zinc and NT-3.

There was significant positive correlation between serum Zn with GAP-43 levels among epileptic children (*r* = 0.381, *p* = 0.006, Fig. 1A), with lack of significant correlation between serum zinc and NT-3 (*r* = 0.212, *p* = 0.139, Fig. 1B). There were no significant correlation between serum GAP-43 levels with TAC (*r* = 0.056, *p* = 0.701).

### Characteristic Performance of GAP-43 in Diagnosing Epilepsy Among Pediatric Patients

**Fig.2:**
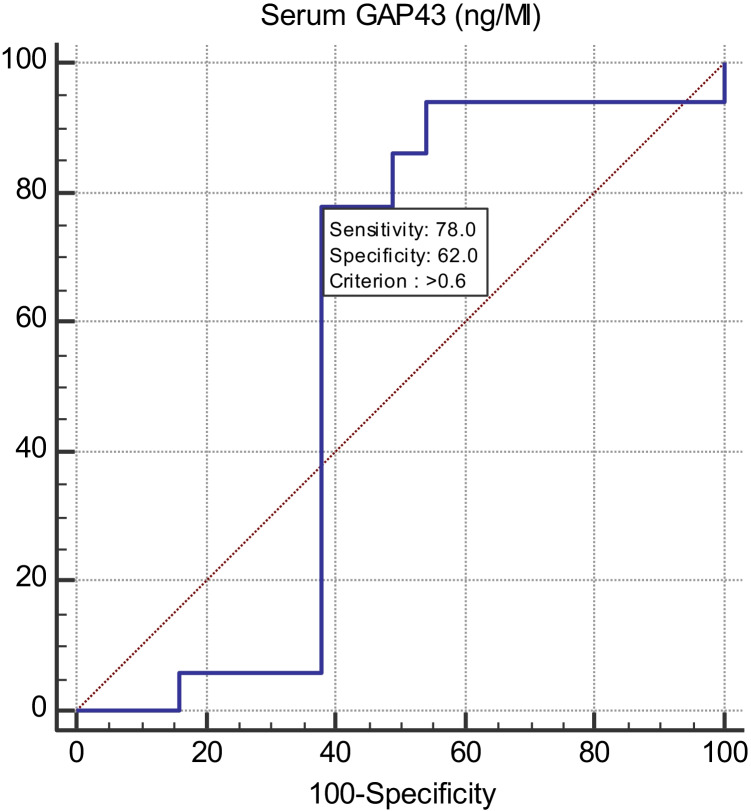
ROC Characteristic Curve for serum GAP43 (ng/mL) for diagnosis of childhood epilepsy

The area under the curve (*AUC*) for GAP-43 was calculated using receiver operative characteristic (ROC) curve to determine the cut-off value for diagnosing FS. The cut-off value could be considered when the parameter’s area under curve (*AUC*) is close to one. As regards the performance characteristics of GAP-43 in diagnosing epilepsy at cut-off point ≤ 0.6 ng/mL showed 78% sensitivity, 62% specificity, positive predictive value (*PPV*) = 50.6%, negative predictive value (*NPP*) = 84.9% with *AUC* = 0.574 (Table 4 and Fig. 2).

**Table 4:** Characteristic performance of serum GAP-43 in diagnosing childhood epilepsy

Cut-off	Sensitivity	Specificity	+ PV	-PV	Accuracy	AUC
≤ 0.6 (ng/mL)	78	62	50.6	84.9	70	0.574

## Discussion

Infants and children are more susceptible to seizures and epilepsy than any other age group. Epilepsies are the most common condition seen in most pediatric neurology clinics in many regions of the developing globe, according to studies. Epilepsy is more common in developing nations, with an incidence of more than 10 per 1000 documented [23]. The prevalence was 6.98 per 1000 in Egypt [24].

According to recent studies, the highest incidence occurs in the first year of life, with a rate of 102/100,000 cases per year, similar to the age range of 1 to 12; the incidence rate in children aged 11 to 17 years old is 21–24/100,000 cases per year [25]. In the present study, the included patients were at the preschool age. This was in agreement with Farghaly et al. [24]. The most common age, according to our findings, was in the early years of life. The lower seizure threshold of the immature brain, which may be due in part to paradoxical excitation of GABA during early brain development, while GABA causes hypopolarization and inhibition of neurons in adulthood, could explain the high incidence of seizure onset during infancy and early childhood compared to late childhood [26].

Regarding parental consanguinity among pediatric patients with epilepsy, our results showed significantly frequent positive consanguinity among patients' parents than controls group. High rate of parental consanguinity was also reported by Kandil et al. [27]. These findings are also due to the fact that consanguineous marriage is more common in rural Egypt than in urban regions. Our findings, on the other hand, contradict those of Saleh et al. [28], who observed a low rate of positive consanguinity in their study.

In terms of epilepsy family history, the included epileptic children had a significant prevalence of epilepsy in their families. Similarly, earlier research has found that having a family history of epilepsy is a significant risk factor for developing epilepsy in children [2, 29–32]. A family history of epilepsy or a history of neurologic or developmental abnormalities may assist narrow the differential diagnosis [33].

In terms of epilepsy clinical types, our findings demonstrated that generalized onset seizures were far more common among epileptic children than simple partial type seizures. Other research in Egypt, Arab countries, and African countries found similar outcomes. Focal epilepsies, on the other hand, are more common in children than generalized epilepsies according to the majority of prevalence surveys from affluent nations [34].The most common type of epileptic seizures, according to Saeed et al. [35], was generalized tonic–clonic seizures. This low occurrence of absence epilepsy may be due to the fact that absence seizures often go unreported and are difficult to detect. Generalized tonic–clonic seizures are the most common type of epilepsy in children, a reflection of the under-identification of other seizure types, most likely due to a lack of recognition and diagnostic tools. This variation can be explained by the number of people at risk, the accuracy of the diagnosis, the distribution of the underlying causes, and whether or not acute symptomatic seizures are included or excluded [36].

There was a significant delay in physical and mental development among epileptic children in the current study, which agrees with Bednark and his colleagues who reported that developmental delay can be associated with epilepsy, which could be the cause of the delay in acquisitions or just one additional manifestation [37].

Clinical research relies heavily on the development of biomarkers for disease diagnosis and treatment. GAP-43 is only found in the nervous system and is preferentially found in excitatory neurons, implying that it has a role in epileptogenesis [38, 39]. Neuroprotection and a marginal regenerative response are the only positive effects of CNS damage. Neurons and glia release cytokines and neurotrophic factors in response to damage, which can activate growth-associated proteins like GAP-43, promoting neuroprotection and regeneration. Furthermore, an increase in GAP-43 was linked to optogenetic-induced functional recovery in the primary motor cortex after a stroke. GAP-43 performs its physiological functions in neurodevelopment, synapse function, and nerve regeneration via altering actin dynamics [40, 41]. Epileptogenesis, the process of brain injury leading to chronic epilepsy, could be regarded as a model of network plasticity [42]. In comparison to the controls, epileptic children had considerably decreased GAP-43 levels. Young rats showed a decrease in GAP-43 mRNA levels in polymorphic areas following a seizure that lasted from 10 h to 3 days, according to an animal model of brain plasticity [43]. Ying et al. [44], on the other hand, found that GAP-43 expression is higher in the epileptic cortex than in the non-epileptic cortex in patients who had surgical brain resection at the Cleveland Clinic Epilepsy Center. Also, Nemes et al. [39] found that cortical dysplasia rats with spontaneous seizures had considerably increased blood GAP-43 levels, implying that GAP-43 is a critical component in epileptogenesis. This discrepancy could be explained by different patient selection criteria, as previously mentioned studies focused on status epilepticus and pharmacoresistant epilepsy (i.e., patients who had taken antiepileptic drugs), which were excluded from our study to avoid any potential confounding effects of antiepileptic drugs. In addition, there were few human investigations; the majority of the studies in the literature were conducted on animal models of epilepsy. Larger-scale researches are needed to confirm our findings.

A decreasing level of NT-3 was observed in an animal model of epileptogenesis, which was followed by the onset of epileptic episodes [45, 46]. Furthermore, after epilepsy, transit alterations in NT-3 expression have been reported [47], implying that NT-3 signalling is involved during seizure attack. Furthermore, evidence from animal models strongly supports the idea that long-term anti-epileptic medication treatment lowers NT-3 levels [48], showing the critical involvement of NT-3 function in the suppression of epileptic episodes. Our data demonstrated that epileptic children had greater median NT-3 levels than controls, but not to a significant level. This could be due to a small sample size, which has to be proven through larger-scale studies.

To minimize oxidative stress in the brain, various antioxidative systems collaborate. The brain’s principal antioxidative mechanisms are nonenzymatic antioxidants such as serum albumin, serum bilirubin, and uric acid, vitamin E, and microelements (selenium and zinc), as well as enzymatic antioxidative mechanisms such as superoxide dismutase and glutathione peroxidase [49]. The individual’s total antioxidant capacity includes both enzymatic and nonenzymatic endogenous antioxidants. These antioxidant mechanisms scavenge oxygen and nitrogen free radicals [50]. The etiology of neuronal hyperexcitability and seizures has been linked to excessive generation of free oxygen and nitrogen radicals [50]. As evidenced in our study by significantly higher TAC and lower zinc serum levels among pediatric patients with newly diagnosed epilepsy compared to healthy controls, epileptic seizures induced oxidant generation stimulate the body’s antioxidant defense mechanisms in an attempt to balance the oxidants’ effect, resulting in high TAC serum levels with consumption of antioxidant trace elements such as zinc. In line with our findings, Eissa et al. [51] reported significant lower levels of serum zinc in a sample of Egyptian epileptic children. Similary, Farahani et al. [52] and Talat et al. [53] reported similar results. Low zinc levels in the blood may increase seizure activity by blocking the inhibitory neurotransmitter GABA or by causing superoxide dismutase and glutathione peroxidase synthesis to be deficient. Hypozincemia stimulates *N*-methyl-d-aspartate (NMDA) receptors, which may play a role in epileptic discharge induction [54].

To the best of our knowledge, we firstly explore a significantly positive correlation between serum zinc and GAP-43 levels among epileptic children. Furthermore, our findings suggest that serum GAP-43 can be used as a good negative biomarker for excluding epilepsy in children when combined with clinical correlation, as the results of our study on the characteristic performance of serum GAP-43 in diagnosing epilepsy revealed that GAP-43 was more sensitive than specific at cut-off point ≤ 0.6 ng/mL, with a higher negative predictive value than positive predictive value.

## Conclusion

GAP-43 may have a key part in the pathogenesis of epilepsy in children, and it can be utilized to rule out epilepsy in children who experience seizures. In addition, epileptic children's antioxidant systems are activated resulting in consumption of antioxidant trace elements such as zinc, which helps to combat the free radicals generated during seizures.

## Study’s Limitations

There were no serial GAP-43 and NT-3 assays available post-seizures to see if their concentrations are largely related to the degree of neuronal injury or recovery. In addition, there were no assays in the CSF samples of the epileptic children included in the study, which could better reflect the pathogenic role of the biomarkers investigated in childhood epilepsy. There was no long-term follow-up of the epileptic children included in the study to correlate the examined markers with the disease’s clinical history. Also, more in-depth accurate assays e.g., RT-PCR to be used in future research to confirm the findings of the current study.

## Data Availability

The datasets used and analyzed in this study are available upon reasonable request.
